# Methods of the Analysis of Oxylipins in Biological Samples

**DOI:** 10.3390/molecules25020349

**Published:** 2020-01-15

**Authors:** Ivan Liakh, Alicja Pakiet, Tomasz Sledzinski, Adriana Mika

**Affiliations:** 1 Department of Pharmaceutical Biochemistry, Medical University of Gdansk, Debinki 1, 80-211 Gdansk, Poland; liakh_ivan@mail.ru (I.L.); tsledz@gumed.edu.pl (T.S.); 2 Department of Environmental Analysis, Faculty of Chemistry, University of Gdansk, Wita Stwosza 63, 80-308 Gdansk, Poland; alicjapakiet@gmail.com

**Keywords:** oxylipins, biological samples, HPLC, UHPLC, GC–MS, LC–MS

## Abstract

Oxylipins are derivatives of polyunsaturated fatty acids and due to their important and diverse functions in the body, they have become a popular subject of studies. The main challenge for researchers is their low stability and often very low concentration in samples. Therefore, in recent years there have been developments in the extraction and analysis methods of oxylipins. New approaches in extraction methods were described in our previous review. In turn, the old analysis methods have been replaced by new approaches based on mass spectrometry (MS) coupled with liquid chromatography (LC) and gas chromatography (GC), and the best of these methods allow hundreds of oxylipins to be quantitatively identified. This review presents comparative and comprehensive information on the progress of various methods used by various authors to achieve the best results in the analysis of oxylipins in biological samples.

## 1. Introduction

Oxylipins are important lipid mediators that are formed from polyunsaturated fatty acids (PUFAs) such as arachidonic acid (ARA), linoleic acid (LA), α-linolenic acid (ALA), eicosapentaenoic acid (EPA), and docosahexaenoic acid (DHA) [1,2,3] in reactions catalyzed by cyclooxygenase (COX), lipoxygenase (LOX), and cytochrome P450 (CYP 450) enzymes, and non-enzymatic oxidation pathways [1,2]. Oxylipins are involved in various biological processes but, primarily, are important for the regulation of inflammation [4,5,6,7,8]. The direction of oxylipins influence on inflammation depends on their PUFA precursor, usually n-3 PUFA derived oxylipins are anti-inflammatory and pro-resolving [1], while n-6 PUFA metabolites can promote inflammation [1,3]. The ability of oxylipins to act as molecular mediator arises from their binding to peroxisome proliferator-activated receptors (PPARs) and G protein-coupled receptors (GPCRs) [9,10]. In epithelial-derived cancers altered ARA metabolism by COX and LOX leads to production of pro-inflammatory factors that promote tumor growth and facilitate formation of tumor microenvironment conductive to angiogenesis and immunosuppression [6,10]. Altered PUFA metabolism was also shown in obesity [4], where the interaction with PPAR and GPCR can modulate adipogenesis [9]. Plasma oxylipin levels have also been correlated with outcome of cardiovascular disease, metabolic syndrome, preeclampsia, due to vasoconstrictive effects of some oxylipins, or cardiac arrythmias [11].

The presence of most oxylipins in low concentrations, as well as their enormous heterogeneity and the emergence of many structurally similar oxylipins, makes their qualitative and quantitative determination difficult due to the low sensitivity of traditional methods. In the previous review [12], we described the current methods of sample preparation from various biological materials preceding the analysis of oxylipins. This paper included a description of the stages of sample collection and storage and a summary of the used pre-extraction additives and standards. These are especially important to consider in order to minimize oxidation, photodegradation or heat destruction of oxylipins during sample handling. Furthermore, the main extraction methods including protein precipitation (PPT), liquid-liquid extraction (LLE), solid-phase extraction (SPE), and the derivatization process were described. Choosing appropriate extraction method and solvents is essential in obtaining good target analyte recoveries and reproducibility needed for further quantitative analysis and depends on the oxylipin group of interest. SPE, due to the availability of various types of sorbents and solvents, is the most widely used extraction method in the analysis of oxylipins. For LLE and SPE, the extraction methods have been grouped according to the characteristics of the studied biological material (biofluids, solid tissues, cell cultures). Finally, new approaches and trends in material collection (dried blood spot), precipitation (ferromagnetic particle enhanced deproteination), and extraction (microextraction, online SPE, mixed-mode extraction with a spin column) for the analysis of oxylipins were described. Owing to the fact that oxylipins are usually present at very low concentrations in biological samples, the quality of sample collection, storage and extraction methods is paramount for achieving accurate quantification. The present review, being an extension of the previous paper, describes further stages of analysis and the quantitative determination of oxylipin levels in the prepared samples using existing analytical techniques.

**Figure 1:**
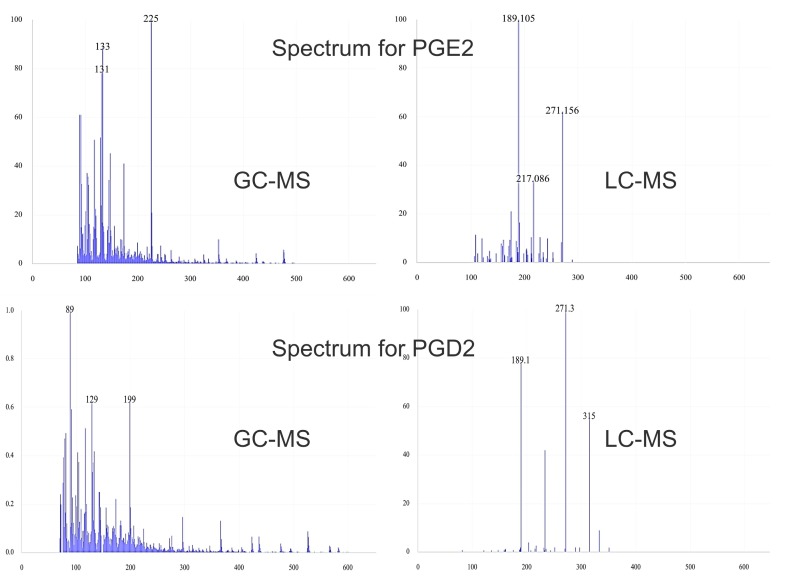
Representative mass spectra of prostaglandin E2 (PGE2) and prostaglandin D2 (PGD2) obtained by using hard (gas chromatography–mass spectrometry (GC–MS)) and soft ionization (liquid chromatography–mass spectrometry (LC–MS)/MS) in spectrometry mass.

Immunoassay, thin layer chromatography (TLC), HPLC with a diode array or fluorescent detector and capillary electrophoresis with a photodiode array detector were used to analyze oxylipins [13,14,15,16,17,18,19]. However, a very similar structure, limited stability and extremely low concentrations of oxylipins in the tissues impose some restrictions on these methods. Therefore, recently, gas chromatography–mass spectrometry (GC–MS) and liquid chromatography–mass spectrometry (LC–MS) have most often been used to determine oxylipin levels in biological samples [20,21,22,23,24]. A summary of the most frequently used analytical methods in the analysis of oxylipins as well as their advantages and limitations is presented in Table 1. Also, the comparison of mass spectra of PGE2 and PGD2 obtained by two most popular techniques for lipids identification: GC–MS and LC–MS is presented on Figure 1 [25].

## 2. Immunoassay Methods

For a long time, immunoassays (enzyme immunoassay (EIA) and radioimmunoassay (RIA)), due to their high sensitivity, were the most widely used quantitative techniques for oxylipins: RIA has been developed to quantify concentrations of IsoPs (8-iso-PGF2α) in human plasma and urine [26], 15-keto-dihydro-PGF2α in human plasma [62], Prostaglandin E2 (PGE2) and LTB4 in human prostate tissues [63], PGE2 in human plasma [64,65], PGE2, PGF2α, PGI2, 6-oxo-PGF2α, TXA2, TXB2 [66] and PGF2α, PGI2, TXA2, 13,14-dihydro-15-keto PGF2α (M-PGF2α), 6-keto PGF1α, and TXB2 [67] in human follicular fluid. ELISA, similar to EIA and RIA, requires specific antibodies, and due to the structural similarity of oxylipins, it is unlikely that antibodies that will sufficiently distinguish them can be obtained [68]. RIA and EIA are sensitive enough to measure subpicomole amounts of oxylipins, but have some limitations for tissue and plasma samples, which diminishes immunoassay sensitivity. Plasma proteins can bind to eicosanoids, and there is a significant degree of immunological cross-reactivity among commercially available eicosanoid antibodies (e.g., the PGE2 antibody can cross-react to a significant extent with PGE3 and 8-iso-PGF2a) [69,70]. To solve these problems, and to separate eicosanoids from plasma proteins, LLE or SPE is used, and the chromatographic separation of eicosanoids is necessary to avoid immunological cross-reactivity [71]. Miller et al. showed that oxylipin levels determined by ELISA correlate well with the levels determined by RIA (LTC4 and LTB4) and HPLC (LTC4) [30]. However, later Schmidt et al. showed the advantages of LC–ESI–MS/MS over previously used immunoassays in the determination of PGE2 and PGD2 in rat microdialysis samples [72]. Also, among the shortcomings of these types of analysis, can be noted a lack of specificity for complex biological fluids, and immunoassays are designed to determine only single metabolites [73]. Also, Gandhi et al. found that the LC–MS/MS method offers a cost-effective and more sensitive alternative to ELISA for the quantification of PGE2, PGD2, PGF2α, LTB4 and TXB2 in rat brain and spinal cord tissues [74]. Henkel et al. compared EIA and LC–MS/MS to determine LTs, PGE2, and TXB2 in human macrophage supernatants and found that EIA showed both inflated levels and higher analyte variability compared to LC–MS/MS [75]. Thus, due to these disadvantages of immunoassay, in recent years, LC–MS/MS has largely replaced this method for determining oxylipin levels.

## 3. Mass Spectrometry Coupled with Separation Techniques

### 3.1. Separation Techniques

#### 3.1.1. High-Performance Liquid Chromatography

In recent decades, HPLC has been the main technique of LC-analysis for the separation of oxylipins, and has superseded TLC. However, conventional analysis using HPLC with UV detection is a difficult task, due to the fact that the conjugated dienes and keto groups absorb at a low UV range (235–280 nm), and most of the oxylipin molecules do not have suitable chromophores, thus the sensitivity/selectivity is insufficient for their analysis in complex extracts from cells or tissue [69,76]. However, UV absorption can be taken into account to further confirm the LC–MS measurement results [77]. For these reasons, a plasma that contains high levels of numerous UV-absorbing compounds is not suitable for determining eicosanoids, the concentrations of which in plasma are lower, as well as PGs, which do not absorb UV light [47]. Since these compounds do not contain aromatic or natural fluorescent systems, GC–MS and, likewise, HPLC combined with fluorescent detectors requires these compounds to be derivatized into a complex that fluoresces, which makes the analysis time-consuming and expensive [50,78]. Also, HPLC with electrochemical detection allows the determination of picogram amounts of lipoxin (LX) A4 (LXA4) and LXB4 in extracts of human polymorphonuclear granulocytes [79], and leukotriene (LT) B4 (LTB4) from human polymorphonuclear leukocytes [80]. However, this method is only suitable for the determination of electrochemically active substances, to which most oxylipins do not apply, in this case additional transformations of substances are required [81,82]. In the HPLC method, the use of various stationary phases allows the use of different modes of separation of oxylipins—reversed-phase (RP)–HPLC, normal-phase (NP)–HPLC, chiral HPLC and hydrophilic interaction chromatography (HILIC). Separation in RP–HPLC relies on the hydrophobic properties of the analytes and therefore remains the main method for the separation of the metabolites of arachidonic acid (ARA) [24]. The RP mode performed mainly on octadecyl silica (C18) or octyl silica (C8) columns provides the highest selectivity for the resolution of isobaric oxylipins [83]. Also, Chen et al., when comparing the chromatographic characteristics of an HSS T3 C18 column and a core-shell C30 column, showed the great potential of the latter in the separation of lipid molecules [84]. The use of a core-shell column instead of a standard C18 column is promising from the point of view of reducing analysis time and improving the chromatographic resolution of PGs [85]. In addition, in terms of the stability of stationary phases, core–shell or fused-core columns are comparable to the conventional columns used in UHPLC but have a lower back pressure (350 bar) [86].

Optimization of chromatographic separation can be achieved by changing the mobile phase composition, gradient conditions, stationary phases and run time. However, some compounds such as IsoP, nitro fatty acids, prostaglandins (PGs), sphingoids, and lysophosphatidic acid species in biological samples are difficult to separate in a single chromatographic run under normal conditions. Schumann et al. solved this problem using two runs: with low pH (between 3.2 and 3.5) and with high pH (between 8.5 and 10.3) [87]. Chromatographic separation also depends on the chemical structure of the compounds. Aoyagi et al. found that the position of the hydroxyl and epoxy groups in the acyl chains of oxylipins can influence the retention time of LC, while epoxy-containing oxylipins elute later than hydroxyl-containing ones. In addition, oxylipins in which the hydroxyl or epoxy group is located at the end of the acyl chain elute earlier than others [88]. Chromatographic separation is usually achieved using gradient elution, which is obtained by mixing solvents A and B. 0.1% acetic acid (HAc) or 0.1% formic acid (FAc) (rarely phosphoric acid) in water (*v*/*v*) is commonly used as solvent A; solvent B can consist of different solvents: Acetonitrile (ACN), isopropanol (IPA), methanol (MeOH), individually or mixed in different proportions (e.g., ACN/IPA–90/10, 70/30 or 50/50 v/v; ACN/MeOH–80/15 *v*/*v*). Also, solvents A and B may contain ammonium acetate and ammonium formate as additives. By the addition of additives (HAc (0.02 and 0.05%), FAc (0.02 and 0.05%), 5mM ammonium formate and 5 mM ammonium acetate) in solvent A, Berkecz et al. studied the effects of solvent on the retention times of oxylipins from human plasma [83]. Using HAc and FAc at 0.05% showed the highest retention times compared to ammonium acetate where the lowest retention was observed. Using FAc and HAc without any additive allowed higher chromatographic resolution to be obtained for isomers. However, for ALA, γ-linolenic acid, 5-hydroxyeicosatetraenoic acid (HETE), 11,12-epoxyeicosatrienoic acid (EET), 15-HETE, 5,15-dihydroxyeicosatetraenoic acid (DiHETE), 6-trans LTB4, 11-HETE, 12-HETE, 4-hydroxydo-cosahexaenoic acid (HDHA) and 14-HDHA, mobile phases with ammonium acetate demonstrate better results [84]. Shaik et al. found that water and ACN mobile phases with HAc provide optimal conditions when measuring prostanoids in rat brain. They also obtained a 2-fold increase in peak area and sensitivity with the addition of 15 μL of 1% (*v*/*v*) HAc in methanol to the eluent before sample evaporation [89]. Also, for the analysis of oxylipins, a technique such as ultra-high performance supercritical fluid chromatography (UHPSF) was successfully used. To improve the separation of oxidized phospholipids such as hydroxides, epoxides and hydroperoxides of linoleic acid and arachidonic acid, Uchikata et al. established an analytical system using supercritical fluid chromatography on a 2-ethylpyridine column. Oxidized phosphatidylcholine isomers from mice liver were separated and further detected using the MRM method with SFC/MS/MS, as RP chromatography cannot solve this problem [90]. But in another study, Berkecz et al. compared the methods UHPSFC/ESI–MS with UHPLC/ESI–MS and found that the sensitivity of UHPLC/MS for the determination of oxylipins is higher (except for prostaglandins) [83].

#### 3.1.2. Other Separation Techniques

##### Chiral Chromatography

One of the problems in the analysis of oxylipins is the presence of critical separation pairs (compounds that have an identical molecular composition, similar fragmentation and close retention time), as well as a large number of isomers that can be formed during non-enzymatic oxidation. To solve this problem, several alternative solutions exist. One of them is the use of chiral chromatography and the application of special chiral columns (Immobilized Polysaccharide or Protein-Based columns) [91]. Deems et al. used an LC system with a Chiralpak AD-H derivatized amylose column (250 × 4.6 mm; Chiral Technologies, West Chester, PA) coupled with MS to isolate eight pairs of isomeric eicosanoids from a cell culture medium [92]. Oh et al. used a Chiralpak AD-RH column (150 × 2.1 mm, 5 μm; Chiral Technologies, West Chester, PA, USA) for the chiral HPLC–MS–MS lipidomic profiling of 18-R/S-HEPEs and 18R/S-resolvin (Rv) E2 stereoisomer pairs from human sera [93,94]. This approach allowed for identification of novel EPA-derived 18S resolvins, which were not apparent without chiral separation, and, upon analyzing biological samples, identification of 18S-HEPE as precursor to this series of resolvins. Later, Deng et al. used this method (LC–MS/MS with a Chiralpak AD-RH column) to study the formation of 14R-HDHA and 14S-HDHA in human macrophages incubated with 12-LOX and identification of novel 13R,14S-diHDHA—maresin 2 [95]. Mesaros et al. used a chiral LC/ECAPCI–MS method with a Chiralpak AD-H column (250×4.6 mm, 5 μm; Daicel Chemical Industries Ltd., Tokyo, Japan) for the analysis of six EET enantiomers formed in urine and tissue samples [96]. Kolmert et al., using a Chiralpak AD-RH analytical column (150 × 2.1 mm, 5 μm; Daicel Collaboration, France), achieved the separation of 13 chiral oxylipins from a human bronchus during an LC–MS/MS analysis [97]. Thus, the use of chiral chromatography is critical not only for separation of known isomer pairs or groups, but allows for the identification of novel compounds, which may poses interesting bioactivity, such as maresin 2, which displayed both anti-inflammatory and pro-resolving action [95]. The disadvantages of chiral chromatography include the need for long-term equilibration, the use of an isocratic gradient and thus a longer analysis time [35,74,98,99], and the use of barely accessible internal standards [100].

Another solution for the separation of isomer pairs is the use of a smaller particle size (UHPLC) or core-shell material columns [86], and more careful optimization of the gradient conditions and solvent composition [101]. Gouveia-Figueira et al. [102] used a Waters BEH C18 column (150 × 2.1 mm, 2.5 μm) during a UHPLC–ESI–MS/MS analysis of oxylipins in human plasma to separate critical pairs of isomers (Resolvin D1/D2 and PGE2/PGD2). In order to obtain the best results, they tried different compositions and gradients of the mobile phase [102]. Using a Zorbax Eclipse Plus C18 column with a diameter of particles less than 2 microns (150 × 2.1 mm, 1.8 μm; Agilent, Waldbronn, Germany) and an optimized gradient, Kutzner et al. separated the maresin (Mar)1 and 7 (S)-Mar1 and protectin (N)PD1 and PDX stereoisomers from human serum and the results were comparable to those achieved by using a chiral column [103]. The high resolution power of approaches using these columns is usually used for separation of known isomers, not for identification of novel compounds.

Finally, isomer pairs were analyzed using combination of both above mentioned approaches. Fuchs et al. combined UHPLC–MS/MS and chiral LC–MS to investigate the stereochemistry of trihydroxyoctadecenoic acids (TriHOMEs) in human BALF samples. They used a UHPLC BEH C18 column (150 × 2.1 mm, 1.7 µm; Waters, Milford, MA, USA) for the rapid quantification of diastereomers and a Chiralpak AD-RH column (150 × 2.1 mm, 3 µm; Daicel, Illkirch, France) for the relative abundance of all stereoisomers. As a result, for the first time, the authors were able to measure all 16 TriHOME isomers in a single chromatographic run. [35]. Also, to identify HETEs/EETs isomers (16-HETE, 17-HETE, and 18-HETE), Chen et al. used a unique heatmap-assisted strategy which included excluding the most abundant common critical pair ions, and a heatmap analysis of unique fragment ions and transitions [84]. Thus, chiral chromatography effectively separates the isomers of oxylipins; however, due to some disadvantages (see Table 1), it is more often used for the targeted analysis of specific oxylipins.

##### Ion Mobility Spectrometry

Ion mobility (IMS) is another separation technique which can be used to improve identification and characterization of oxylipins in biological samples, especially separation of isomers. The IMS implementation between chromatographic and MS step provides another dimension of separation, increasing certainty of oxylipin identification-addition of collision-cross-section (CCS) value. This approach has been successfully used by Kyle et al. [104] for separation of 42 isomer pairs or groups in either positive or negative mode, for example *R* and *S* isomers of HETEs; better separation was obtained for sodiated [M + Na]^+^ ions rather than deprotonated species [M − H]^−^. A variation of IMS was also used by Hinz et al. [105]. In this study ions were travelling through drift tube filled with low pressure nitrogen gas in which their drift time was affected by ion size and shape, which allowed for formation of different oxylipin conformers. However, the use of IMS requires building additional CSS libraries, use of either computationally generated standards, or literature CCS [105], which complicates implementation of this method.

##### Immunoaffinity Column Chromatography

Immunoaffinity chromatography (IAC) is a type of LC in which the stationary phase consists of an antibody or synthetic protein-binding reagent, and it is a highly efficient method to isolate a particular compound from biological samples for measurement by GC–MS or LC–MS [106]. IAC is highly selective and specific for the identification and quantification of prostanes, isoprostanes (IsoP) and their metabolites, which commonly occur at very low concentrations in biological fluids such as plasma and urine [107]. However, there is a limitation to IAC as it is exclusively commercialized for the analysis of 15-F2t-isoprostane and not for other metabolites of n-3 and n-6 PUFAs [2]. Gijon et al. showed the possibility of using immunoaffinity extraction enrichment (IAE) to improve the analysis of certain lipids present in trace amounts in certain test samples. The use of this double extraction protocol for the extraction of leukotrienes (LTs) gave such advantages, when compared to traditional SPEs, as minimal risk of column overload, cleaner samples, and the flow-through fluid can be used to analyze compounds not retained by the antibodies [108]. Tsikas et al. used Sepharose 4-based IAC columns (4-mL, 1-mL gel resin; Cayman Chemicals, Ann Arbor, MI, USA) for the extraction and GC–tandem mass spectrometric (MS/MS) quantification of prostaglandin (PG) E1 (PGE1) in human plasma [42]. However, the lack of ready-made antibodies, in combination with the complexity of antibody production methods, limits the widespread use of IAC [106].

##### Thin-Layer Chromatography

TLC, popular in the last century, can be used as an additional sample preparation technique before MS analysis. Liu et al. developed a method including SPE, TLC purification, chemical derivatization, and GC–MS detection for the quantification of F2-IsoPs from a variety of biological sources [38]. Also, TLC was applied to separate thromboxane (TX) B2 (TXB2) and 11-Dehydro-TXB2 in human plasma [109] and for the quantitation of 15-F2t-IsoP in rat plasma [39]. Moreover, Tsikas et al. compared IAC (4-mL (1-mL gel resin) 8-Isoprostane Affinity Column) with TLC for the quantification of 8-iso-PGF2α in human urine. The combination of TLC followed by quantification by GC–MS yields twice as high values for 8-iso-PGF than the combination of IAC extraction with GC–MS [107].

### 3.2. GC–MS

Maximum development in the study of oxylipin levels by GC–MS took place in the 1980s. For GC analysis, the molecule must be volatile and thermally stable, which is not the case with oxylipins; as a result, the analysis requires the derivatization of carboxyl and hydroxyl groups to increase their volatility [14]. Reagents such as N, O-bis(trimethylsilyl)-trifluoroacetamide (BSTFA) are used for the silylation of hydroxyl groups, with consequent detection in electron impact (EI) mode [110]. To increase the sensitivity of the analysis, GC is almost always coupled with MS detection, which allows multiple analytes to be detected in one sample, greatly reducing the cost of routine detection [47]. The GC–MS technique has wide application in the analysis of oxylipins in urine and plasma samples. Tsikas notes the widespread use of GC–MS in the quantitative measurement of PGs, TXs, LTs, IsoPs, and other ARA metabolites in human urine [55]. Later Tsikas and Zoerne reviewed various reports of research on eicosanoids in plasma, serum, and other biological fluids of healthy humans, measured by validated GC–MS, GC–MS/MS, and LC–MS/MS methods. GC–MS allows the analysis of such different compounds as LTs (LTB4), TXs (TXB2, 11-dh-TXB2), prostacyclins (6keto–PGF1α), prostaglandins (PGF2α, PGE1, PGE2, PGD2), and F2-IsoPs (15(S)-8-iso-PGF2α) [21]. The authors also note that most of the reported LC–MS/MS methods have several-fold higher lower limits of quantitation (LLOQ) values than the reported GC–MS/MS methods, which is the undoubted disadvantage of LC–MS/MS [2,21]. At the same time, Puppolo et al. summarized analytical methods for ARA and its metabolites in the brain, and showed that GC–MS is widely used for PGE2, PGD2, PGF2α, 8,9-dihydroxyeicosatrienoic acid (DiHETrE), 5,6-DiHETrE, 12-HHT, 2-HETE, 3-HETE, 5-HETE, 8,9-HETE, 11,12-HETE, 15-HETE, 8-iso-PGF2α, 9α,11β-PGF2α, 9α,11 β-PGF2α, PGE2, PGD2, TXB2, PGF1α, PGF2α, F2-IsoP, and ARA quantification in brain tissue [24].

GC–MS is widely used for the quantification of F2-IsoPs from a variety of biological sources [2,39,111,112,113]. Liu et al. validated the methodology to quantify F2-IsoPs in biological fluids and tissues using GC–MS that includes SPE, TLC purification, derivatization, and MS detection using negative ion chemical ionization (NICI) [38]. At the same time, GC–MS methods allow all possible F2-IsoPs stereoisomers to be quantified, while LC–MS methods permit the separation and identification of selected regioisomers and diastereomers of F2-IsoPs [38]. Also GC–MS was used as an alternative to enzyme-linked immunosorbent assay (ELISA) for the quantification of IsoPs in human urine and plasma [114], because results from ELISA and GC–MS cannot be compared (ELISA overestimates urinary 15-F2t-IsoP concentrations) [31,115]. Milne et al. used GC–MS to simultaneously quantify IsoPs and isofurans (IsoFs). While numerous methodologies for the quantification of F2-IsoPs have been presented in the literature, including alternate GC/MS based assays as well as LC/MS assays and enzyme immunoassays [116], no alternative methodologies to quantify IsoFs have been reported. Also GC–MS was used for the measurement of F2-IsoP in rat liver plasma and urine [32]. GC–MS analysis has been widely used to estimate other types of oxylipins in various types of biological tissues. Margalit et al. used GC–MS for the assessment of 14 biologically significant eicosanoids (ARA, PGE2, PGE2,-d4, PGD2, PGF2α, PGE1, 6-Keto PGF1α, TXB2, hepoxilin A3 (HxA3), 12-HPETE, 12-hydroxyeicosatrienoic acid (HETrE), 12-HETE, 15-HETE, LTB4, LTC4) in rat air pouches and human whole blood [117]. Tsukamoto et al. used GC–MS for the simultaneous quantification of PGE2, PGD2, PGF2α, 8-epi-PGF2α, 6-keto-PGF1α, TXB2 from a cell-cultured medium (RAW264.7 and U937 cells) [118]. Also GC–MS was used for the determination of peripheral plasma prostanoid concentrations (PGE2, PGF2α, 6-keto-PGF1α and TXB2) [119,120], PGE1, PGE0 and 15-keto-PGE0 in human plasma [121], LTB4 in human serum [122], and the analysis of 11-dehydro-TXB2 in human urine [123] and plasma [109]. Nithipatikom et al. used GC–MS/NICI to analyze EETs, DHETs and 20-HETE in coronary venous plasma during coronary artery occlusion and reperfusion in dogs [124]. Werner et al. used GC–MS after the purification of cell cultures by RP–HPLC to investigate the formation of dihydroxyeicosatetraenoic acids (DHETs) and HETEs (5,6-DHET, 8,9-DHET, 11,12-DHET, 14,15-DHET, 5-HETE, 8-HETE, 9-HETE, 11-HETE, 12-HETE, and 15-HETE) in human peritoneal macrophages [125].

### 3.3. LC–MS/MS

Since LC–MS/MS is the most frequently chosen technique for analyzing oxylipins (a more detailed description of the used conditions and analytical tools is presented in Table 2), in this section we would like to draw attention to the difficulties encountered in LC–MS/MS analysis, and the methods used by different authors to eliminate them. 

One of the main advantages of LC–MS/MS is that derivatization is not required, which prevents impurities from being introduced and increases sensitivity in MS analysis, as well as reducing time and cost [24]. Tandem MS/MS instruments coupled with HPLC or ultra-high performance liquid chromatography (UHPLC) are capable of analyzing multiple analytes simultaneously [150]. The main two scanning modes of an MS/MS instrument are multiple reaction monitoring (MRM) and selected reaction monitoring (SRM) modes. While in scanning mode specific ranges of mass are studied in the first or second analyzer, in MRM mode product ions are produced from precursor ions after the collision-induced dissociation (CID) which increases the specificity of the analysis [24] and allows the identification of oxylipins with structural similarity [140]. Using identification only by the retention time and SRM transition of the analytes makes difficult the fact that oxylipins can be represented by several isomers. Detection in SRM mode can help in the case where LC cannot separate 8 and 12-HETE, and they coelute. Conversely, 9 and 12-HETE have similar MS–MS spectra and they must be separated chromatographically [150], as well as in the case of PGD2 and PGE2 [151] and 8-iso-PGF2 and PGF2 [86], which have identical patterns of fragmentation. Also, to maximize the specificity and sensitivity of oxylipin analysis, tandem mass spectrometry (MS/MS) with a triple quadrupole (QqQ) detector in MRM mode can be used in the case of co-eluting metabolites [140].

Presently, targeted metabolomic LC–MS approaches for ARA metabolites allow the simultaneous quantification of more than 100 oxylipins with high sensitivity (LOD 0.01–0.21 pg on the column) in a run time of about 25 min. In all of these methods, the RP LC is connected to a highly sensitive QqQ MS system operating in negative electrospray ionization (ESI) mode [150]. A combination of UHPLC chromatographic separation and MRM transitions performed on a QqQ allowed 184 eicosanoid metabolites to be separated and quantified in a 5-min running time [60].

When coupling LC with MS, the solvent selection is important as mobile phase additives and buffers can lead to ion suppression. In most of the mobile phases, weak acids are used for the analysis of oxidized PUFA species by LC–MS (e.g., 0.1% FAc or HAc), preventing the formation of carboxylate anions in the ESI source [74]. Also, HAc enhanced the ESI (-) ionization of plasma oxylipins compared to the mobile phase without any additive (ca. 90% on average for 0.02%, and 40% for 0.05% of HAc), while the addition of ammonium acetate or formate resulted in a drastic loss of sensitivity [83]. Golovko and Murphy found that using different brands of methyl formate during tissue extraction before PG LC–MS analysis led to very high chemical background noise, accounting for a 5- to 20-fold reduction in sensitivity for different PGs compared with acetone LLE [152]. Chen et al., when optimizing the ionization processes (MS signal), compared various additives (HAc, FA, ammonium formate) in composition with the same mobile phase (50/50, ACN/H_2_O), and it was shown that 0.1% FA showed less variation and higher average responses [84].

LC–MS analysis not only requires the highest level of purity of the reactants, but also additional purification steps of the sample (PPT, LLE, SPE) to eliminate the effect of the matrix components and reduce the matrix effect [56,60,74,153]. The matrix effect is the suppression or enhancement of the ionization of analytes by the presence of matrix components in biological samples [154]. Matrix effects occur when molecules co-eluting with the analytes alter the ionization efficiency of the electrospray interface. Matrix effects are also compound-dependent; the most polar compound was found to have the largest ion suppression, and the least polar was affected less by ion suppression. The interfering matrix components may come from the current sample, a previously injected sample or the overload of the analytical column [155]. To most effectively remove or minimize the influence of matrix effects, the following improvements can be used: modifications to the sample extraction methodology, improved chromatographic separation, and using stable isotope-labeled internal standards (IS) [154]. Deuterium-labeled standards may have disadvantages such as different retention times as compared to analytes, undesired amplification or the weakening of ionization, while ^13^C-labeled standards theoretically may be better for analysis but they are not commercially available [37,156]. The choice of the optimal amount of IS is also important, since the interaction of the analyte/internal standard affects the accuracy [157,158]. For the efficient removal of the ESI interfering matrix in plasma, special SPE procedures are undertaken using anion-exchange stationary phases (oxylipin carboxy acid moiety) or non-polar (water) and polar (n-hexane) washing stages before the elution of oxylipins [150]. If the IS does not compensate for all matrix effects, it is possible to use the IS with a more heavy isotope label, which will increase the accuracy and reliability of oxylipin analysis [150], and it should also be remembered that, despite the fact that most non-certified standards are good quality, it is recommended to calculate correction factors in order to adjust concentrations and compensate for differences during their use [78]. In addition, in order to reduce the effect of the phospholipid-based matrix in LC–ESI–MS, the matrix can be diluted as far as possible before loading it into the sorbent, and a slightly higher concentration of organic modifier used for washing and elution can be taken. [159]. To obtain low matrix effects and a good yield of extraction, Dupuy et al. optimized the sample preparation and the extraction process of iso-prostanoids from human plasma, which included Folch extraction, basic hydrolysis and SPE (Oasis Max, 60 mg; Waters) purification [142]. Also, using a short UHPLC column containing very small particles (50 × 2.1 mm, 1.7 µm), compared to a HPLC column (100 × 3.0 mm, 3.5 µm), for the quantification of seven urinary eicosanoid species, allowed Sterz et al. to obtain shorter run-times and sharper peaks and thus improved signal-to-noise ratios [98].

The matrix effect also depends on the type of tissue being examined, and how complex and rich the matrix is [87]. For colon tissue, the matrix effect was very important with a signal loss of about 50% for PUFA metabolites [135], in contrast to the determination of urinary levels of oxylipins (LTB4) when the matrix effect led to a 10% signal loss [160], or the matrix effect was absent for urinary eicosanoids [98] and oxylipins [161].

UHPLC increases the resolution, speed and sensitivity of the analysis of metabolites, and additionally, lowers the use of solvent, which decreases costs [24]. The application of columns 2-μm less instead of the traditional LC columns has significantly improved the chromatographic separation of eicosanoids in human plasma and reduced the analysis time from 20~60 min to 4–12 min [84]. Kortz et al. compared a standard C18 column (C18, 250 × 2.1 mm, 5 μm; Vydac, Grace Vydac) and a Kinetex core-shell column (C18, 100 mm ×  2.1 mm, 2.6 μm; Kinetex, Phenomenex) for the LC–ESI–MS/MS (5500 QTrap; AB SCIEX) analysis of standard mixtures of 15 eicosanoids. Using a Kinetex core-shell column at a flow rate of 0.5 mL/min (P = 315 bar) improved the resolution of PGD2 and PGE2 from 1.4 to 2.9 and reduced the analysis time by half [85].

Brose et al. tested reverse-phase UHPLC columns like: BEH C18 (150 × 2.1 mm,1.7 μm), BEH HILIC (100×2.1mm, 1.7 μm), ACQUITY (Waters, Milford, MA, USA), CSH C18 (150 × 2.1 mm, 1.7 μm), HSS T3 (150 × 2.1 mm, 1.8 μm) and HSS C18 (150 × 2.1 mm, 1.8 μm). They used gradients with acidified ACN/water, MeOH/IPA/water, and MeOH/water, to quantify PGs (Q-TOF, Synapt G2-S; Waters, Milford, MA) with the ESI source. All commercially available PGD2- and PGE2 like iso-PG was separated on the HSS T3 column (ACN/water acidified with 0.1% FAc gradient); it was also possible to separate brain endogenous iso-PG (except for PGE2/ent-PGE2 and 8iso-PGE2/15R-PGE2). That allowed 5 times sharper peaks to be obtained compared to the previously used LC–MS/MS method, with a fast 4 min separation [162]. Gouveia-Figueira et al. elaborated and validated a novel UHPLC–ESI–MS/MS method for the analysis of the oxylipins in human plasma. Using a Waters BEH C18 column (150 × 2.1 mm, 2.5 μm) coupled with an Agilent 6490 Triple Quadrupole system with the iFunnel Technology source (Agilent Technologies, Santa Clara, CA, USA), they achieved the separation of 37 oxylipins, (including some critical pairs of isomers) [102]. Song et al. developed a UHPLC–MRM/MS platform for the simultaneous determination of 122 eicosanoids in human whole blood (HWB). An AB SCIEX API 4000 system (AB SCIEX, Foster City, CA, USA) was used for MS detection. Using UHPLC separation with a sub-2-μm column (150 × 2.1 mm, 1.7 µm, Kinetex C18 column; Phenomenex) led to a total run time reduction to 6.5 min (including equilibration of the column) without the loss of peak resolution or detection sensitivity. The average peak width narrowed to 3 s with a UHPLC/sub-2-μm column from 9 s compared to a conventional HPLC column. Also, injecting only 5 μL of the sample obtained from 2.5 μL of HWB (which consists of 1/4 of the volume needed for the conventional HPLC protocol) showed the equivalent sensitivity [137].

Mass spectrometric optimization also plays a big role and can lead to a significant gain in the signal and thus lower LLOQ values. Various parameters such as source temperature (ST), collision energy (CE), declustering potential (DP), collision cell exit potential (CEP), collision activated dissociation (CAD), and gas pressure affect the signal intensity in the SRM mode. Simultaneously, caution should be taken with increasing the temperature of nebulizer gas as well as auxiliary gas, because the optimum temperature for some compounds may lead to the thermal degradation of others [103]. To obtain better detection limits and avoid interference from nearby isomers, at the last step of optimization, the most intense and specific fragments and transitions are usually chosen [101,103,163]. However, in the case of interference ions, it is better to select more characteristic rather than stronger fragmentation ions. For example, when Yang et al. developed a method for murine sera and BALF oxylipin profiling, for 8, 9 EET the weaker transition 319.2/123 was chosen, because the stronger transition 319.2/167 overlaps with the transition 319.2/167 for 11, 12 EET [101]. Also, to increase the sensibility of the MS method, the optimization of MS operating conditions should be carried out for each individual compound [15,69,103,131,164]. By the careful selection of transitions, it is possible to optimize sensitivity and selectivity so that the overall sensitivity will improve several times [85,101].

Sometimes, simple operations can significantly improve the performance of the analysis. Kolmert et al. determined 109 analytes from human and guinea pig lung tissue using the LC−MS/MS method. They showed that the average recovery increased by 13% by adding 30 μL of 30% glycerol (in MeOH) before eluate evaporation. In addition, even washing the inner surface of the tube with a reconstitution solvent for 10 s increased recovery by 19% [97]. Mengesha also showed that the use of nitrogen gas at room temperature in a vacuum dryer for pre-concentration leads to a decrease in the signals of eicosanoids, with an especially high loss of analytes with a large proportion of ACN, while adding other solvents to ACN reduced the loss of analytes [158]. Also, the organic composition of the mixture for sample re-dissolution can be modified by adding 10–30% water to methanol; this does not affect the solubility of hydrophobic analytes but improves the peak shape for polar analytes [97]. After the optimization of MS operating conditions (DP, CE, CEP, CAD), Kutzner et al. [103] achieved a significant signal gain. Additionally, the increase of the injection volume to 10 μL resulted in lower LLOQ values. A further increase in injection volume to 20 μL was possible only when the extract of the sample was dissolved in 1:1 MeOH/water, because the recovery of the extract in pure organic solvent led to an unacceptable peak shape [103].

### 3.4. Mass Spectrometry—“Shotgun” Lipidomics

There are two fundamentally different approaches for mass spectrometry-based lipidomic analyses. One approach—comprehensive lipidomic analysis by separation simplification (CLASS), is based on the separation of different lipid categories using optimal extraction and chromatographic techniques prior to mass analysis [165]. The second approach (shotgun lipidomics) is based on the ability of ESI to analyze multiple components in the sample simultaneously, which allows the profiling of many lipid classes in biological tissue in parallel, wherein the sample is introduced into the mass spectrometer without separation, and fragmented components are scanned in MS/MS modes [163]. Wang et al. used the direct infusion of human plasma after LLE and one-step derivatization for the structural identification of the composition of eicosanoid isomers by using ESI–MS/MS [166]. Also, Milic et al. described a new derivatization technique for the simultaneous detection of the oxidation products of DHA, ARA, LA, and oleic acid by ESI–MS in positive ion mode [167]. And if this approach causes difficulties (isobaric species, ion suppression) in complex matrices as plasma, such simple matrices as urine are devoid of this problem [168]. However, in most cases, when analyzing oxylipins an absolute quantitation is required, which needs prior separation [129].

## 4. Oxylipin Identification/Annotation

Further analysis of oxylipins, depending on the purpose of the study, can be carried out using two main approaches. The first “targeted approach” includes targeted analysis of specific types of oxylipins. Most often, for positive identification, the MRM of the analyte as well as the retention time (in order to differentiate analytes with identical MRM transitions) are compared with those of the standard. Usually for each analyte, after single injections, unique transitions are selected that are additionally compared with the literature. Scanning “transitions” on a QqQ provides a sensitive quantitative analysis, but this approach is limited by the number of available commercial standards, so most published methods analyze only a small fraction of the known oxylipins.

To obtain high-quality lipidomic information about all the main molecular species of oxylipins in the sample, the second approach, namely “untargeted” (non-targeted), is used. In this procedure, non-targeted lipidomics are performed using high-resolution MS (mainly QTOF) and all the detected ions are identified using a database based on fragmentation values. Recently, Watrous et al. developed a new approach for using non-targeted LC–MS/MS to classify putative known oxylipins. Based on the fragmentation patterns obtained from a large number of standards, they maintained a network due to which it was possible to classify more than 500 different oxylipins in human plasma [169]. It is also possible to combine both approaches like Wheelock et al., who used targeted (QqQ) metabolomics to identify 36 oxylipins and non-targeted (orbitrap) to annotate 219 metabolites [170].

Oxylipin researchers currently work with a large number of databases in manual or automatic mode to identify compounds based on their mass spectra. Among the most popular are the following databases: The LIPIDMAPS open source lipid database, which, based on mass generation by computational methods, allows the search for existing masses in the LIPID MAPS Structure Database (LMSD), the calculation of the exact mass of lipid ions, the prediction of MS/MS spectra, and also shows such structural properties as the exact mass, formula, abbreviation, etc. [171,172]. The LipidBlast open source computer-generated (in-silico) tandem mass spectral (MS/MS) lipid database, which contains about 8000 unique structures [173]. The Lipid Mass Spectrum Analysis Database (LMSAD), which is a database containing MS data, structures and annotations of biologically significant lipids [174]. The LipidHome database of theoretical lipid structures providing theoretically generated lipid molecules [175]. LipidBank, the official open source lipid database of the Japanese Conference on the Biochemistry of Lipids (JCBL) [176,177]. The Human Metabolome Database (HMDB), which is a freely accessible electronic database containing information on the metabolites of small molecules found in the human body [178]. MassBank—a storage of mass spectral data that includes several thousands of lipids and lipid metabolites [179]. The METLIN database, obtained by the individual analysis of substances using MS/MS data generated on various types of devices [180]. In oxylipin analysis also databases can be used such as MINE databases [181], LipiDAT, [182], NIST [183], CYBERLIPID [184], LipidPedia [185], and omicX databases for lipidomics analysis (https://omictools.com), and the American Oil Chemistry Society (AOCS) lipid library (https://lipidlibrary.aocs.org).

### Programs and Tools

To simplify and automate the process of searching data in known databases special software is often used. These tools allow not only for automatic identification from open databases and an in-house library, but also the quantification of lipids according to LC–MS data. Many of them allow the primary MS/MS raw data received from various devices to be directly exported, and often contain statistics modules for the analysis of results. To minimize the possibility of errors, these programs are usually equipped with various filters to eliminate unwanted matches; some of them allow the use of rule-based identification. Below, are the most popular commercial and free available ones used for these purposes: Lipid View [186], Lipid Search [177], SimLipid [187], LipidXplorer [188], LipiDex [189], LIMSA [190], Lipidyzer [191], Lipid Data Analyzer [192], LipidQA [193], CEU Mass Mediator [194], LipidLama [195], LipidMatch [196], LipidMiner [197], dGOT-MS [198], ALEX [199], The Lipid Annotation Service (LAS) [200], and LOBSTAHS [201]. Thus, the data accumulated over many years make the non-targeted approach simpler, and therefore more popular among researchers. At the same time, the traditional targeted approach will remain indispensable for the quantification of oxylipin levels.

## 5. Conclusions

Among the various analytical tools, tandem MS/MS instruments have recently become the most popular due to their high sensitivity for analyzing the levels of oxylipins in biological samples, while UHPLC offers the highest resolution, speed and sensitivity for analyzing oxylipins. The development of instrumentation makes operation easier, speeds up analysis, and provides improved selectivity and sensitivity, and lower detection limits.

## Figures and Tables

**Table 1:** Advantages and limitations of the most frequently used analytical methods in the analysis of oxylipins.

Analytical Technique	Application	Advantages	Limitations
Immunoassay methods	Eicosanoids, IsoPs, LTs, TxBs, PGs	Enzyme immunoassay (EIA) and dioimmunoassay (RIA) are easy to use, sensitive and do not require expensive instrumentation [2,26]	Unspecificity due to cross-reactivity [27]
		RIA combined with HPLC can greatly enhance sensitivity [28]	Limited to a single metabolite at a time [29]
		ELISA is comparable to RIA and HPLC in sensitivity and selectivity [30]	Target only a few compounds [30]
			Metabolite overestimation due to cross-reactivity [31,32]
			RIA requires long analysis run times and generates hazardous waste [33]
Chiral chromatography	HETEs, PGs, EETs, TriHOMEs, Maresins, Protectins, Lipoxins	Allows the enantiomeric separation of oxylipins [34]	Long equilibration and run time, and limited sensitivity [34]
			Reduced sensitivity (with increasing peak width and elution time, the signal/noise decreases) [35]
			Not a high throughput (used for targeted analysis) [36]
			Very sensitive to changes in mobile-phase composition [34,37]
Capillary electrophoresis	EETs, DHETs	Allows the resolution of EET and DHET regioisomers [20]	Limited number of analytes [16]
			Long run time [17]
Thin layer chromatography	IsoPs, PGs, keto-PGs, TXs	Used for the preliminary separation of analytes [38,39,40]	Loss of products at the TLC stages [41]
			Requires large plasma volumes [42]
HPLC-UV or fluorescence detection	PGs, EETs, HETEs, LTs	Provides structural information [43]	Limited number of UV-light absorbing analytes [44]
		Sufficient sensitivity and selectivity [45]	Sensitivity is low [46,47]
			Requires two distinct separation methods to quantify the prostaglandin products and the HETE and leukotriene products [48]
			Long analysis time required [49]
			HPLC with fluorescence detection also requires derivatization [50]
MALDI-TOF/MS	PGs	Allows the direct quantification of prostaglandins at levels similar to LC–MS/MS analysis [51]	Needs the co-crystallization of a matrix with the sample, which affects the quantification of the analytes [52]
		Soft ionization technique allows for the analysis of non-volatile compounds [52]	Needs derivatization steps [51]
		Less sensitive to impurities (buffer salts) compared to other MS methods [53]	
GC-EI/MS	All classes	When coupled with MS detection, GC is both highly sensitive and selective [21,24]	Needs derivatization of the analytes [54]
		Multiple analytes can be detected in one sample [47]	Requires the thermal stability of the analytes [55]
		Higher sensitivity compared to LC–MS/MS [21]	There is no derivatization strategy for some compounds [56,57]
			Absolute requirement of volatility [57]
ESI-MS/MS	All classes	Simple, fast, no sample carry-over [58] (shotgun analysis)	Creates a common product ion spectrum, inability to separate the isomers [59] (shotgun analysis)
		High selectivity and good sensitivity [60]	Ion suppression [15]
			During switching of the ionization mode (negative/positive) a loss of sensitivity occurs [61]

**Table 2:** Analytical methods based on LC–MS used in oxylipin analysis.

Oxylipin	Biological Material	Extraction Method	Analytical Column	Mobile Phases	Instrumental Analysis	Analyzer	LOD	Reference
11-trans-LT (C4, E4,) 15-deoxy-Δ12,14-PG (D2, J2) 6-trans-LT (B4, 12-epi-LTB4) DiHETE (5,6-, 5,15-) DiHETrE (5,6-, 8,9-, 11,12-, 14,15-) EpETrE (5(6)-, 8(9)- , 11(12)-, 14(15)-) HETE (5-, 8-, 9-, 10-, 11-, 12-, 15-, 20-) HHTrE (12-); HpETE (5-, 12-, 15-) LT (B4, C4, E4); LX (A4); Oxo-ETE (5-, 12-) PG (B2, D2, D2-EA, 6-keto-E1, E2, E2-EA, F2α, F2α-EA, J2) PGE2 (bicyclo-, dhk-, 19-hydroxy-, 20-hydroxy-, 15-keto) PGF2α (11β-, dhk-, 2,3- dinor-11β-, 20-hydroxy-, 15-keto-) tetranor (PGEM, PGFM); TX (B2)	cell culture, cell medium	SPE	RPLC: Grace-Vydac C18 2.1 × 250 mm	RPLC: A: H_2_O/ACN/FAc (63/37/0.02, *v*/*v*/*v*) B: ACN/IPA (50/50, *v*/*v*)	LC–MS/MS	QQQ-LIT	1–1000 pg	[92]
			Chiral LC: Chiralpak AD-H 4.6 × 250 mm	Chiral LC: A: Hex/anhydrous ethanol/H_2_O/FAc (96/4/0.08/0.02, *v*/*v*/*v*) B: 100% anhydrous ethanol				
EDP (7,8-, 10,11-, 13,14-, 16,17-, 19,20-) EpETE (5(6)-, 8(9)-, 11(12)-, 14(15)-, 17(18)-) EpETrE (5(6)-, 8(9)-, 11(12)-, 14(15)-) HDoHE (20-); HEPE (20-); HETE (20-)	rat heart, kidney, brain, liver, lung, pancreas, red blood cells, plasma	SPE	Zorbax Eclipse Plus-C18 4.6 × 150 mm, 1.8 μm	A: ACN B: 0.01 M ammonia acetate	HPLC–MS/MS	QQQ	0.05–0.1 ng	[126]
6-keto-PGF1α HETE (5-, 8-, 11-, 12-, 15-) LTB4; PG (D2, E2, F2α); TX (B2)	mouse serum, human lung epithelial cells, rat fibroblast cell line culture medium	SPE, derivatization	Ascentis Express C18 2.1 × 150 mm, 2.7 μm	System I A: H_2_O/ACN/HAc (95/5/1, *v*/*v*/*v*) B: 1% HAc in ACN	LC-ESI-MS/MS	QQQ	–	[127]
				System II A: 0.1% FAc in H_2_O B: 0.1% FAc in ACN				
PG (D2, 15R-PGD2, E2, 11β-PGE2, 8-iso-PGE2)	mice brain	LLE	Luna C18 2.0 × 150 mm, 3 μm	A: 0.1% FAc in H_2_O B: 0.1% FAc in ACN	HPLC-ESI-MS/MS	QQQ	~3 pg	[128]
11-trans-LT (C4, D4, E4) 14,15-LT (C4, D4, E4) 15-deoxy-PG (A2, D2, J2) 15-keto-PG (E1α, E2, F2α) 5-iso-PGF2αVI; 6-keto-PGs (E1, F1α) 8-iso-PGF2αIII; dhk PGs (D2, E2, F2α) DiHETE (5,6-, 5,15-, 8,15-) DiHETrE (5,6-, 8,9-, 11,12-, 14,15-) DiHoHE (10*S*,17*S*-); DiHOME (9,10-, 12,13-) dihomo PG (15-deoxy PGJ2, D2, E2, F2α, J2) EpETE (14(15)-, 17(18)-) EpETrE (5(6)-, 11(12)-, 14(15)-) EpOME (9(10)-, 12(13)-) HDoHE (4-, 7-, 8-, 10-, 11-, 13-, 14-, 16-, 17-, 20-) HEPE (5-, 8-, 9-, 11-, 12-, 15-, 18-) HETE (5-, 8-, 9-, 11-, 12-, 15-, 16-, 17-, 18-, 19-, 20-) HHTrE (12-); HODE (9-, 13) HOTrE (9-, 13-, 13-HOTrE-γ) HX (A3, B3); LT (B4, C4, D4, E4) LX (A4, A5, B4) Oxo-EDE (15-); Oxo-ETE (5-, 15-) Oxo-ODE (9-, 13-); PD1 PG (A2, B2, D1, D2, D3, E1, E2, E3, F1α, F2α, F3α, J2, K2) PGE2 (11β-, 19-hydroxy-, 20-hydroxy-, bicyclo-) PGF1α (6,15-dk-dh-, 17 6-keto-) PGF2α (11β-, 11β-dhk-, 19-hydroxy-, 20-hydroxy-, 2,3-dinor-11β-, dh-) Rv (D1, E1) tetranor (12-HETE, PGEM, PGFM) TXs (B1, B2, B3)	cell medium, rat spinal cord tissue, murine papilloma, murine tibiotarsal ankle joint	SPE	Synergi reverse-phase C18 2.1 × 250 mm	A: H_2_O/ACN/HAc (70/30/0.02, *v*/*v*/*v*) B: ACN/IPA (50/50, *v*/*v*)	LC–MS/MS	QQQ-LIT	0.1–1 pg	[129]
dhk-PG (E2, F2α, D2) DiHDoHE (10,17-) DiHETrE (5,6-, 8,9-, 11,12-, 14,15-) DiHoPE (19,20-) EpETrE (5(6)-, 11(12)-, 14(15)-) HDoHE (17-); HEPE (5-, 12-) HETE (5-, 11-, 12-, 15-, 20-) HHTrE (12-); HODE (13-); LT (B4, D4) PG (E2, F2α, 8-iso-PGF2α, D3, E3) TriHOME (9,10,13-); TX (B2, B3)	mouse plasma, liver, ileum and adipose tissue	SPE	Acquity C18 BEH 2.1 × 100 mm, 1.7 μm	A: 0.1% FAc in H_2_O B: 0.1% FAc in ACN	UHPLC–MS/MS	QQQ	–	[130]
DiHETrE (5,6-, 8,9-, 11,12-, 14,15-) EpETrE (8(9)-, 11(12)-, 14(15)-) HETE (5-, 8-, 9-, 11-, 12-, 15-, 20-) HpETE (5S-); LT (B4, C4, D4, E4) LX (A4); PG (D2, E2, J2, 6-keto-PGF1α, dhk-PGF1α, F2α, 8-iso-PGF2α) tetranor (PGEM, PGFM); TX (B2, 11-dehydro-TXB2, 2,3-dinor-TXB2)	human plasma	SPE	Luna C8 2.0 × 150 mm, 5 μm	A: 0.5 mM ammonium formate in H_2_O (pH 3.3) B: 0.5 mM ammonium formate in ACN (pH 3.3)	HPLC–MS/MS	QQQ-LIT	10–400 pg/mL (LLOQ)	[131]
HETE (12-); LT (E4) PGF2α (2,3-dinor-8-iso-, 8-iso-) tetranor (PGEM); TX (2,3-dinor-TXB2, 11-dehydro-TXB2)	human urine	LLE	BEH C18 2.1 × 50 mm, 1.7 μm	A: 0.1% FAc in H_2_O B: 0.1% FAc in ACN	UHPLC-SRM/MS	QQQ	0.002–0.06 ng/mL urine	[98]
DiHDPA (19,20-); DiHETE (14,15-, 17,18-) DiHETrE (5,6-, 8,9-, 11,12-, 14,15-) DiHOME (9,10-, 12,13-) EpDPE (19(20)-); EpOME (9(10)-, 12(13)-) HEPE (5-, 12-, 15-); HETE (5-, 8-, 11-, 12-, 15-) HETrE (15S-); HODE (9-, 13-) HOTrE (9-); HpODE (9-, 13-) Oxo-ODE (9-, 13-) PG (F2α, dh-PGF2α, F1α, E2, 11β-PGE2) TriHOME (9,10,13-, 9,12,13-); TX (B2)	human plasma	SPE	Ascentis Express 2.1 × 150 mm, 2.7 μm	A: 0.1% HAc in H_2_O B: ACN/IPA (90/10, *v*/*v*)	LC-ESI-MS/MS	QQQ	0.1–11.4 nM	[132]
DiHDPE (4,5-, 7,8-, 10,11-, 13,14-, 16,17-, 19,20-) DiHETE (5,6-, 5,15-, 8,15-, 8,9-, 11,12-, 14,15-, 17,18-) DiHETrE (5,6-, 8,9-, 11,12-, 14,15-) DiHODE (9,10-, 12,13-, 15,16-) DiHOME (9,10-, 12,13-) EpDPE (7(8)-, 10(11)-, 13(14)-, 16(17)-, 19(20)-) EpETE (8(9)-, 11(12)-, 14(15)-, 17(18)-) EpETrE (5(6)-, 8(9)-, 11(12)-, 14(15)-) EpODE (9(10)-, 12(13)-, 15(16)-) EpOME (9(10)-, 12(13)-) HDoHE (17-); HEPE (5-, 8-, 12-, 15-) HETE (5-, 8-, 9-, 11-, 12-, 15-, 20) HETrE (15S-); HODE (9-, 13-) HOTrE (9-, 13-); LT (B3, B4, B5) LTB4 (6-trans-, 20-OH-, 20-COOH-) LX (A4); Oxo-ETE (5-, 12-, 15-) Oxo-ODE (9-) PG (D1, E1, B2, D2, E2, J2, 15-deoxy-PGJ2, D3, E3, F2α) RvE1; TriHETrE (10,12,15-) TriHOME (9,12,13-, 9,10,13-); TX (B2)	human plasma	SPE	Eclipse Plus C18 2.1 × 150, 1.8 μm	A: 0.1% glacial HAc in H_2_O B: 0.1% glacial HAc in ACN/MeOH (84/16, *v*/*v*)	LC–MS/MS	QQQ-LIT	–	[133]
2,3-dinor-6- keto-PGF1α TX (B2, 2,3-dinor-TXB2)	human urine	SPE, derivatization	Acquity UHPLC BEH C18 2.1 × 150 mm, 1.7 μm	A: 0.1% HAc in H_2_O B: ACN/IPA (90/10, *v*/*v*)	UHPLC–MS/MS	QQQ	0.85–15.2 fmol	[134]
2,3-dinor-6-keto-PGF1α 8,12-iso-iPF2α-VI dhk-PG (E1, E2, F2α) PG (D2, E1, E2, F2α); PGF2α (2,3-dinor-8-iso-, 2,3-dinor-11β-, 8-iso-, 11β-) tetranors (PGDM, PGEM)		SPE					0.55–15.4 fmol	
14,15-LT (C4, D4, E4) LT (B4, 6-trans-LTB4, C4, D4, E4,)		SPE		A: 0.2% FAc in H_2_O B: 0.2% FAc in ACN/IPA (90:10, *v*/*v*)			3.02–4.59 fmol	
11β-PGs (F2α, E2); 14,15-LT (C4, E4) 15-deoxy-Δ12.14-PG (D2, J2) 15-keto-PG (E2, F2α) 20-hydroxy-PG (F2α, E2) 6-keto-PG (E1, F1α) 8-iso-15-keto-PG (E2, F2α) 8-iso-PG (A2, E2, E3, F2α, F3α) dhk-PG (F2α, E2, tetranor-F1α, D2) DiHDoHe (10S,17S-) DiHETE (5,6-, 8,15-, 5,15-)DiHETrE (5,6-, 8,9-, 11,12-, 14,15-) EpETrE (5(6)-, 8(9)-, 11(12)-, 14(15)-) HEPE (5-, 11-, 12-) HETE (5-, 9-, 11-, 12-, 15-, 16-, 17-, 18-, 19-, 20-) HHTrE (12-); HpETE (5-, 12-, 15-) iPF2α-IV; LT (B4, C4, E4, D4) LTB4 (20-hydroxy-, 15,15-dehydro-, 18-carboxy-dinor-, 20-carboxy-, 12-oxo-); LX (5S,6R-LXA4, 5S,14R-LXB4) Mar1; Oxo-ETE (5-, 12-, 15-) PG (A2, B2, D1, D2, D3, E1, E2, E3, F1α, F2α, F3α, J2) PGF2α (11β-dhk-, 2,3-dinor-11β-, 2,3-dinor-8-iso-); Rv (D1)tetranor (12-HETE, PGFM, PGEM, PGDM) TX (B1, B2, 11-dehydro-TXB2, B3)	human plasma	online SPE	Kinetex C18 2.1 × 100 mm, 2.6 μm	A: H_2_O/ACN/FAc (63/37/0.02, *v*/*v*/*v*) B: IPA/ACN (50/50, *v*/*v*)	online-SPE-LC–MS/MS system	QQQ-LIT	–	[86]
DiHETE (5,6-) EpETrE (5(6)-, 8(9)-, 11(12)-, 14(15)-) HDoHE (14-, 17-); HEPE (18-) HETE (5-, 8-, 12-, 15-, 19-, 20-) LT (B4, B5); LX (A4, B4) Mar1 (7-); Oxo-ETE (5-); PDx PG (E2, E3, 6-keto-PGF1α) Rv (D1); TX (B2)	mouse colon tissue, human epithelial colorectal adenocarcinoma cells supernatant, foam macrophages supernatant, mouse peritoneal exudate	SPE	ZorBAX SB-C18 2.1 × 50 mm, 1.8 μm	A: H_2_O/ACN/FAc (75/25/0.1, *v*/*v*/*v*) B: ACN/FAc (100:0.1, *v*/*v*)	HPLC–MS/MS	QQQ	0.06–15.63 ng/mL	[135]
DiHDPE (10,11-, 13,14-, 16,17-, 19,20-) DiHETE (11,12-, 14,15-, 17,18-) DiHETrE (8,9-, 11,12-, 14,15-) DiHODE (9,10-, 12,13-, 15,16-) DiHOME (9,10-, 12,13-) EpDPE (10(11)-, 13(14)-, 16(17)-, 19(20)-) EpETE (11(12)-, 14(15)-, 17(18)-) EpETrE (8(9)-, 11(12)-, 14(15)-) EpODE (9(10)-, 12(13)-, 15(16)-) EpOME (9(10)-, 12(13)-) HEPE (5-, 12-, 15-); HETE (5-, 8-, 9-, 11-, 12-, 15-) HODE (9-, 13-); HOTrE (9-, 13-)	human serum	SPE	Zorbax Eclipse Plus C18 2.1 × 150 mm, 1.7 μm	A: 0.1% HAc in H_2_O B: ACN/MeOH/HAc (80/15/0.1, *v*/*v*/*v*)	LC-ESI-MS	QQQ-LIT	0.25–7 nM vial (LLOQ)	[136]
11-trans-LT (C4, E4, D4) 14,15-LT (C4, E4,) 15-deoxy-PG (A2, D2, J2) 6-keto-PG (E1, F1α) dhk PG (PGF2α, E2, D2) DiHDPA (19,20-) DiHETE (5,6-, 5,15-, 8,15-) DiHETrE (5,6- 8,9-, 11,12-, 14,15-) DiHOME (9,10-, 12,13-) EpDPE (16(17)-, 19(20)-) EpETE (14(15)-, 17(18)-) EpETrE (5(6)-, 8(9)-, 11(12)-, 14(15)-) EpOME (9(10)-, 12(13)-) HDoHE (4-, 7-, 8-, 10-, 11-, 13-, 14-, 16-, 17-, 20-) HEPE (5-, 8-, 11-, 12-, 15-, 18) HETE (5-, 8S-, 8R-, 9-, 11-, 15-, 16-, 17-, 18-, 19-, 20-) HETγE (5-, 8-, 15-); HHTrE (12-)HODE (9-, 13-); HOTγE (13-, 13-HOTγEg, 9-) LT (B4, C4, E4, D4) LTB4 (12-epi, 12-oxo-, 20-carboxy, 20-hydroxy-, 6-trans-, 6-trans-12-epi-) LX (14R-LXA4, A4, A5, B4)Oxo-EDE (15-); Oxo-ETE (5-, 12-, 15-) Oxo-ODE (9-, 13-); PD1PG (A2, B2, D1, D2, D3, E1, E2, E3, F1α, F2α, F3α, J2, K2) PGE2 (11β-, 19-hydroxy-, 15-keto-, 20-hydroxy-, bicyclo) PGF1α (15-keto-, 6,15-dk-dh-) PGF2α (11β-, 11β-dhk-, 15-keto-, 19-hydroxy-, 20-hydroxy-, 5-isoPGF2αVI, 8-iso PGF2αIII) PGF2α (Δ17 6-keto-, 2,3-dinor-11β-, dh-, dihomo-) Rv (D1, D2) tetranor (12-HETE, PGEM, PGDM) TX (B1, B2, B3)	human whole blood	SPE	Kinetex C18 2.1 × 150 mm, 1.7 μm	A: H_2_O/ACN/HAc (70/30/0.1, *v*/*v*/*v*) B: IPA/ACN/HAc (50/50/0.02, *v*/*v*/*v*)	UHPLC–MS/MS	QQQ	–	[137]
DiHDPA (19,20-) DiHETrE (5,6-, 8,9-, 11,12-, 14,15-) DiHOME (9,10-, 12,13-) EpOME (12(13)-) HDoHE (4-, 7-, 10-, 11-, 13-, 14-, 16-, 17-, 20-) HEPE (5-, 12-, 18-) HETE (5-, 8-, 11-, 12-, 15-, 16-, 18-) HETrE (15-); HODE (9-, 13-) HOTrE (9-, 13-); MarOxo-ODE (13-) PG (D2, E2, 6-keto-PGF1α, F2α) TriHOME (9,10,13-, 9,12,13-); TX (B2)	human plasma, rat kidney	SPE	Luna C18 2.0 × 250 mm, 5 μm	A: H_2_O/ACN/FAc (70/30/0.02, *v*/*v*/*v*) B: IPA/ACN (50:50, *v*/*v*)	LC–MS/MS	QQQ-LIT	–	[138,139]
11β-PG (E2, F2α, dhk-PGF2α) 15-deoxy-PG (J2, D2, A2) 6-keto-PG (E1, -2,3-dinor-F1α) 8-iso-PG (F3α, F2αIII, 8-iso-15-keto PGF2β) dhk-PG (D2, E2, F2α); DiHDPA (19,20-) DiHETE (5,6-, 5,15-, 8,15-) DiHETrE (5,6-, 8,9-, 11,12-, 14,15-) DiHOME (9,10-, 12,13-) EpDPE (16(17)-, 19(20)-) EpETE (14(15)-, 17(18)-) EpETrE (8(9)-, 11(12)-, 14(15)-) EpOME (9(10)-, 12(13)-) HDoHE (4-, 7-, 8-, 10-, 11-, 13-, 14-, 16-, 17-) HEPE (5-, 8-, 9-, 11-, 12-, 15-) HETE (5-, 8-, 9-, 11-, 12-, 15-, 18-, 19-, 20-) HETrE (5-, 8-, 15-); HHTrE (12-) HODE (9-,13-); HOTrE (13-, 13-HOTrEγ) HX (A3, B3); LT (B4, E4) LTB4 (12-epi, 12-oxo-, 20-carboxy, 20-hydroxy-, 6-trans-, 6-trans-12-epi-) LTE4 (11-trans-, 14,15-) LX (B4, 15R-LXA4, 6S-LXA4, 6R-LXA4, LXA5) Mar1 (17R-); Oxo-EDE (15-) Oxo-ETE (5-, 12-, 15-) Oxo-ODE (9-, 13-); PD (10S-, 15-trans-) PG (A2, B2, D1, D2, D3, E1, E2, E3, F1α, F2α, F3α, J2, K2) PGE2 (19-hydroxy, 20-hydroxy, bicyclo) PGF2α (2,3-dinor-11β-, 2,3-dinor-8-iso-, 20-hydroxy-, 5-iso-PGF2αVI, dh-) Rv (D1, E1) TX (B1, B2, B3, 11-dehydro-TXB2, 2,3-dinor-TXB2)	control human plasma, mouse and human tissue: adipose, liver, muscle	SPE	Acquity UHPLC BEH shield RP18 2.1 × 100 mm, 1.7 μm	A: ACN/H_2_O/HAc (60/40/0.02, *v*/*v*/*v*) B: ACN/IPA (50/50, *v*/*v*)	UHPLC–MS/MS	QQQ-LIT	3–300 pg (LOQ)	[60]
DiHDPE (4,5-, 7,8-, 10,11-, 13,14-, 16,17-, 19,20-) DiHETE (5,6-, 5,15-, 8,15-, 8,9-, 11,12-, 14,15-, 17,18-) DiHETrE (5,6-, 8,9-, 11,12-, 14,15-) DiHODE (9,10-, 12,13-, 15,16-) DiHOME (9,10-, 12,13-) EKODE; EpDPE (10(11)-, 13(14)-, 16(17)-, 19(20)-) EpETE (8(9)-, 11(12)-, 14(15)-, 11(12)-) EpETrE (5(6)-, 8(9)-, 11(12)-, 14(15)-) EpODE (9(10)-, 12(13)-, 15(16)-)EpOME (9(10)-, 12(13)-) HEPE (5-, 8-, 12-, 15-) HETE (5-, 8-, 9-, 11-, 12-, 15-, 20-) HETrE (15S-); HODE (9-, 13-) HOTrE (9-, 13-); LT (B3, B4, B5) LTB4 (20-carboxy-, 20-hydroxy-, 6-trans-); LX (A4) Oxo-ETE (5-, 15-); Oxo-ODE (9-, 13-) PG (B2, D1, D2, D3, E1, E2, E3, 6-keto-PGF1α, F2α, J2, 15-deoxy-PGJ2)Rv (E1); THF diolTriHETrE (1,12,15-) TriHOME (9,10,13-, 9,12,13-); TX (B2)	human plasma	SPE, LLE	Agilent Zorbax Eclipse Plus C-18 2.1 × 150 mm, 1.8 μm	A: 0.1% HAc in H_2_O B: ACN/MeOH/HAc (800/150/1, *v*/*v*/*v*)	LC–MS/MS	QQQ-LIT	0.1–1.30 nM	[23]
DiHETE (5,6-) DiHETrE (11,12-, 14,15-) EpETrE (11(12)-) HETE (5-, 12-, 15-, 20-) LT (B4, 6-trans-LTB4, C4, D4, E4) LX (A4); Oxo-ETE (5-) PG (D2, E2, J2, 15-deoxy-Δ12,14-PGJ2) PGE2 (20-hydroxy-, 15-keto-); TX (B2)	mouse hypothalmus	SPE	Supelco C18 3.0 × 100 mm, 2.7 μm	A: H_2_O/ACN/HAc (69.98/20/0.02, *v*/*v*/*v*) B: ACN/IPA (70/30, *v*/*v*)	LC–MS/MS	QQQ	–	[140]
dhk-PG (D2, E2) EpDPE (16(17)-, 19(20)-) EpETE (8(9)-, 11(12)-, 14(15)-, 17(18)-) EpETrE (5(6)-, 8(9)-, 11(12)-, 14(15)-) EpOME (9(10)-, 12(13)-) HDoHE (4-, 7-, 8-, 10-, 11-, 13-, 14-, 16-, 17-, 20-) HEPE (5-, 8-, 9-, 11-, 12-, 15-, 18-) HETE (5-, 8-, 9-, 11-, 12-, 15-, 16-, 17-, 18-); HETrE (5-, 8-, 15-) HODE (9-, 13-); HOTrE (9-, 13-, 13-HOTrEr) LT (B4); LX (A4); Mar1 (7S-) PD1; PG (D2, E2, 6-keto-PGF1α, F2α, 15-deoxy-PGJ2) Rv (17R-RvD1); TX (B2)	mouse lung homogenate	SPE mixed-mode spin column, SPE	Acquity UHPLC BEH C18 2.1 × 100 mm, 1.7 μm	A: H_2_O/1 M ammonium acetate/5 mM phosphoric acid/FAc (990/10/1/1, *v*/*v*/*v*/*v*) B: ACN/IPA/1 M ammonium acetate/FAc (495/495/10/1, *v*/*v*/*v*/*v*)	LC–MS/MS	QQQ	–	[141]
AdA IsoP (ent-7(R,S)-7-F2t-dihomo-IsoP, 17(R,S) -F2t-dihomo-IsoP) ARA IsoP (15-epi-15-F2t-IsoP, 15-F2t-IsoP, 5-F2t-IsoP, 5-epi-5-F2t-IsoP, 2,3-dinor-15-F2t-IsoP, ent-15(R,S)-2,3-dinor-5,6-dihydro-15-F2t-IsoP, d4-15-F2t-IsoP) ALA PhytoP (ent-16-epi-16-F1t-PhytoP, ent-16-F1t-PhytoP, 9-F1t-PhytoP, 9-epi-9-F1t-PhytoP) EPA IsoP (8-F3t-IsoP, 8-epi-8-F3t-IsoP, 5-F3t-IsoP, 5-epi-5-F3t-IsoP) DHA NeuroP (10-F4t-NeuroP, 4(R,S)-F4t-NeuroP, 10-epi-10-F4t-NeuroP, d4-10-epi-10-F4t-NeuroP, d4-10-F4t-NeuroP, d4-4(R,S)-4-F4t-NeuroP)	human: plasma, CSF, mouse: plasma, urine, brain, liver, muscle	SPE, derivatization	Zorbax SB-C18 Rapid Resolution HD 2.1 × 100 mm, 1.8 μm	A: 0.1% FAc in H_2_O B: 0.1% FAc in ACN	LC–MS/MS	QQQ	0.49–15.63 ng/mL	[142]
DiHETE (5,6-) DiHETrE (11,12-, 14,15-) EpETrE (11(12)-) HETE (5-, 12-, 15-, 20-) LT (D4, E4, B4, 6-trans-LTB4, 12-epi-LTB4) LX (A4); Oxo-ETE (5-) PG (D2, E2, J2, 15-deoxy-Δ12,14-PGJ2, 20-hydroxy-PGE2, 15-keto-PGE2) TX (B2)	human plasma	SPE	Ascentis Express C18 30 × 100 mm, 2.7 μm	A: H_2_O/ACN/FAc (69.98/30/0.02, *v*/*v*/*v*) B: ACN/IPA 970/30, *v*/*v*)	LC–MS/MS	QQQ	5.0 ng/mL (LLOQ)	[143]
DiHDPA (19,20-) DiHETrE (5,6-, 8,9-, 11,12-, 14,15-, 17,18-) EpDPE (19(20)-); EpETE (17(18)-) EpETrE (5(6)-, 8(9)-, 11(12)-, 14(15)-) EpOME (9(10)-, 12(13)-) HETE (5-, 11-, 12-, 15-, 19-, 20-) HODE (9-, 13-)PG (6-keto-PGF1α, F2α, 8-iso-PGF2α, E2, D2) TriHOME (9,10,13-, 9,12,13-); TX (B2)	human whole blood, human platelet-rich plasma	SPE	Luna C18 1 × 150 mm, 5 μm	-	HPLC–MS/MS	QQQ	–	[144]
DiHDPE (19,20-) DiHETE (5,6-, 5,15-, 8,15-) DiHETrE (5,6-, 14,15-) DiHETrE (5,6-, 14,15-) DiHOME (12,13-) EpETE (14(15)-) EpETrE (5(6)-, 11(12)-) EpOME (9(10)-, 12(13)-) HDoHE (4-, 7-, 8-, 9-, 10-, 11-, 14-, 17-, 20-); HEPE (5-, 11-, 15-) HETE (5-, 8-, 9-, 11-, 12-, 15-, tetranor-12-); HETrE (5-, 15-) HHTrE (12-); HOTrE (9-, 13-) LT (B4, E4, 6-trans-LTB4) Oxo-ETE (5-, 15-); Oxo-ODE (9-, 13-) PG (A2, B2, D2, 15-deoxy-Δ12,14-PGD2, D3, H2, E2, F2α, J2) PGE2 (11β-, dhk-) PGF1α (6-keto-, 2,3-dinor-6-keto-) PGF2α (8-iso-, dhk-, 15-keto-, 5-iso-PGF2αVI); Rv (D1) tetranor (PGDM); TX (B2)	human plasma	SPE	Acquity UHPLC BEH C18 2.1 × 150 mm, 1.7 μm	A: ACN/H_2_O/HAc (45/55/0.02, *v*/*v*/*v*) B: IPA/ACN (50/50, *v*/*v*)	UHPLC–MS/MS	Q-IM-TOF	–	[83]
			Acquity UPC2 Torus 1-Aminoanthracene 3 × 100 mm, 1.7μm	A: supercritical MeOH B: 0.1% HAc in MeOH	UHPSFC-MS/MS			
DiHETrE (5,6-, 8,9- 11,12-, 14,15-) DiHOME (9,10-, 12,13-) endocannabinoids EpETrE (5(6)-, 8(9)-, 11(12)-, 14(15)-) EpOME (9(10)-, 12(13)-); HEPE (12S-) HETE (5-, 8-, 9-, 11-, 12-, 15-, 20-) HETrE (15S-); HODE (9S-, 13-) LT (B4); Oxo-ETE (5-, 12-, 15-) Oxo-ODE (13-) PG (D2, E2, E2-EA, F2α, F2α-EA) Rv (D1, D2) TriHOME (9,10,13-, 9,12,13-); TX (B2)	cow heart, cow liver, pig/elk/cow brain, human plasma, lung lavage fluid, milk, cell medium	SPE	Waters BEH C18 2.1 × 150 mm, 1.7 μm	A: 0.1% HAc in H_2_O B: ACN/IPA (90/10, *v*/*v*)	UHPLC–MS/MS	QQQ	0.25–533 fg	[102,145,146]
DiHETrE (5,6-, 8,9-, 11,12-, 14,15-) HETE (5-, 8-, 9-, 11-, 12-, 15-, 16-, 18-) HHTrE (12-); Oxo-ETE (5-, 12-) LT (B4); PG (A2, D2, E2, F2α, J2, 6-keto-PGF1α, dhk-PGF2α) tetranor (PGEM) TX (B2, 11-dehydro-TXB2)	human serum, plasma, washed platelets	SPE	Kinetex C8 2.1 × 150 mm, 2.6 μm	A: 0.1% FAc in H_2_O B: 0.1% FAc in ACN	LC–MS/MS	QQQ	–	[147]
DiHOME (9,10-) EpETrE (5(6)-, 8(9)-, 11(12)-) EpDPA (16(17)-, 19(20)-) EpETE (14(15)-); EpOME (9(10)-) HDoHE (4-)HETE (5-, 8-, 9-, 11-, 12-, 15-) HODE (13-); HOTrE (9S-) LT (B4); LX (A4); Oxo-ODE (9-, 13-)	dried blood spot	LE	Eclipse plus C8 2.1 × 100 mm, 1.8 μm	A: 0.05% FAc in ACN/H_2_O (50/50, *v*/*v*) B: 0.05% FAc in ACN	UHPLC–MS/MS	QQQ	1–20 pg/μL	[20]
PG (A1, A2, D1, D2, E1, E2, F1α, 6-keto-PGF1α, F2α, 8-iso-PGF2α, F3α)	human ovarian follicular fluid	LLE	Nano cHiPLC ChromXP C18-CL 0.5 mm × 200 μm, 3 μm 120 Å	A: 0.1% FAc in H_2_O B: 0.1% FAc in ACN	nano LC–MS/MS	QQQ-TOF	–	[148]
			Synergy hydro RP-C18	A: 0.1% FAc in H_2_O B: 0.1% FAc in ACN	LC–MS/MS	QQQ		
10S,17S-diH (AdA, n3 DPA, n6 DPA) 11-dehydro-TX (B2, B3) 2,3-dinor-TX (B1, B2) 7S,17S-diH (n3 DPA) AdA IsoP/F (ent-7(*R*,*S*)-7-F2t-dihomo-IsoP, 17(*R*,*S*)-17-F2t-dihomo-IsoP 1 and 2, 7(*R*,*S*)-ST-Δ8-11-dihomo-isoF, 17(*R*,*S*)-10-epi-SC-Δ15-11-dihomo-IsoF 1 and 2) ALA PhytoP/F (ent-9-F1t-PhytoP, ent-9-epi-F1t-PhytoP, ent-16-epi-16-F1t-PhytoP, ent-16-F1t-PhytoP, 9-L1-PhytoP, ento-16(*R*,*S*)-13-epi-ST-Δ14-9-PhytoF 1 and 2) ARA IsoP (5(*R*,*S*)-5-F2t-IsoP, 15-F2t-IsoP (8-iso-PGF2α), 15(*R*,*S*)-2,3-dinor-15-F2t-isoP) DHA NeuroP/F (4(*R*,*S*)-4-F4t-NeuroP, 10-F4t-NeuroP, 10-epi-10-F4t-NeuroP, 14(*R*,*S*)-14-F4t-NeuroP, 4(*R*,*S*)-ST- Δ5-8-NeuroF) dhk PG (E1, F2α, tetranor PGE2) DiHDPE (4,5-, 7,8-, 10,11-, 13,14-, 16,17-, 19,20-) DiHETE (5,15-, 8,9-, 8,15-, 11,12-, 14,15-, 17,18-) DiHETrE (5,6-, 8,9- 11,12-, 14,15-) DiHODE (9,10-, 12,13-, 15,16-) DiHOME (9,10-, 12,13-) EPA IsoP (5(*R*,*S*)-5-F3t-IsoP, 8-F3t-IsoP, 8-epi-8-F3t-IsoP) EpDPE (10(11)-, 13(14)-, 16(17)-, 19(20)-) EpETE (8(9)-, 11(12)-, 14(15)-, 17(18)-, 12-OH-17(18)-) EpETrE (5(6)-, 8(9)-, 11(12)-, 14(15)-) EpODE (9(10)-, 12(13)-, 15(16)-) EpOME (9(10)-, 12(13)-) HDoHE (4-, 7-, 8-, 10-, 11-, 13-, 14-, 16-, 17-, 20-, 21-, 22-) HEPE (5-, 8-, 9-, 11-, 12-, 15-, 18-, 19-, 20-) HETE (5-, 8-, 9-, 11-, 12-, 15-, 19-, 20-) HETrE (5S-, 15S-); HHTrE (12S-) HODE (9-, 13-); HOTrE (9-,13-) LT (B3, B4, B5) LTB4 (20-carboxy-, 20-hydroxy-, 6-trans-); LX (A4) n6 DPA NeuroP (4(*R*,*S*)-F3t-NeuroPn6) Oxo-ETE (5-, 15-); Oxo-ODE (9-, 13-) PD1; PG (B2, D1, D2, D3, E1, E2, 20-hydroxy-PGE2, F1α, F2α, J2) PGF1α (15-keto-, 6-keto-, Δ17-6-keto-) PGF2α (11β-, dihomo-) PGJ2 (15-deoxy-, Δ12-) Rv (D1, E1, E2, 18R-RvE3, 18S-RvE3) stearic acid (9,10-diH-, 9(10)-ep-) THF diol; TriHETrE (11,12,15-) TX (B1, B2, B3)	human plasma, human colorectal carcinoma cells	SPE	Zorbax Eclipse Plus C18 2.1 × 150 mm, 1.8 μm	A: 0.1% HAc in H_2_O B: ACN/MeOH/HAc (800/150/1, *v*/*v*/*v*)	LC-(ESI-)-MS/MS	QQQ-LIT	0.05–1.0 nM	[15]
dhk-PG (E1, E2, F2α); dh-PG (E1, F2α) HETE (5-, 8-, 11-, 12-, 15-) LT (B4, C4, 14,15-LTC4, D4, E4) PG (B2, D1, D2, D3, E1, E2, E3, F1α, 6-keto-PGF1α, F2α, F3α, J2, 15-deoxy-Δ12,14-PGJ2) PGE2 (20-hydroxy-, 15-keto-) PGF2α (8-iso-, 11β-, 2,3-dinor-8-iso-) tetranor (PGEM, PGFM) TX (B2, B3, 11-dehydro-TXB3)	human serum, sputum, BALF	SPE	Acquity UHPLC BEH shield RP18 2.1 × 150 mm, 1.7 μm	A: 0.1% HAc in H_2_O B: 0.1% HAc in ACN/MeOH (90/10, *v*/*v*)	UHPLC–MS/MS	QQQ-LIT	0.2–1 ng/mL depending on matrix (LLOQ)	[149]
DiHOME (9,10-, 12,13-) EDP (16,17-, 19,20-) EpETE (14(15)-, 17(18)-) EpETrE (5(6)-, 8(9)-, 11(12)-, 14(15)-) EpOME (9(10)-, 12(13)-) HDoHE (4-, 7-, 10-, 14-, 17-) HEPE (18-) HETE (5-, 8-, 9-, 11-, 12-, 15-, 20-) HODE (9-, 13-); HOTrE (9-, 13-) hydroxy-epoxy-, keto-epoxy-octadecenoic acids, LT (B4); LX (A4, B4); Mar1 Oxo-ETE (5-); Oxo-ODE (9-, 13-) PD (X, 1/NPD1) PG (E2, F2α, 8-isoPGF2α) Rv (D1, D2)TriHOME (9,12,13-, 9,10,11-, 9,10,13-) TX (B4)	human plasma	SPE	ZorBAX RRHD Eclipse Plus C18 4.6 × 100 mm, 1.8 μm	A: 12 mM ammonium acetate/HAc (100/0.02, *v*/*v*) B: 12 mM ammonium acetate in ACN/H_2_O/HAc (90/10/0.02, *v*/*v*/*v*)	UHPLC–MS/MS	QQQ-LIT	0.02–1 ng/mL	[13]
11-trans-LT (C4, D4, E4) 11β-PG (F2α, E2); 14,15-LT (C4, D4) 15-deoxy PG (D2, J2); 15-keto-PG (E2, F1α); 6-keto-PG (E1) dhk-PG (D2, E2, F2α) DiHDPA (19,20-) DiHETE (5,6-, 5,15-, 8,15-) DiHETrE (5,6-, 8,9-, 11,12-, 14,15-) DiHOME (9,10-, 12,13-) dihomo-PG (E2, F2α) EpDPE (16(17)-, 19(20)-) EpETE (17(18)-, 14(15)-) EpETrE (5(6)-, 8(9)-, 11(12)-, 14(15)-) EpOME (9(10)-, 12(13)-) HDoHE (4-, 7-, 8-, 10-, 11-, 13-, 14-, 16-, 17-, 20-) HEPE (5-, 8-, 9-, 11-, 12-, 15-, 18-) HETE (5-, 8-, 9-, 11-, 12-, 15-, 16-, 17-, 18-, 19-, 20-) HETrE (5-, 8-, 15-); HHTrE (12-) HODE (9-, 13-) HOTrE (9-, 13-, 13-HOTrEγ) LT (B4, C4, D4, E4) LTB4 (12-oxo-, 20-carboxy-, 20-hydroxy-) LX (B4, A5, 15R-LXA4, 6S-LXA4) Mar1 (7R-); nitrooleate (9-, 10-) Oxo-EDE (15-); Oxo-ETE (5-, 12-, 15-) Oxo-ODE (9-, 13-); PD1 PG (A2, B2, D1, D2, D3, E1, E2, E3, F1α, F2α, 8-iso PGF2αIII, 8-iso-15-keto-PGF2b, F3α, J2, K2) PGEM, PGFM PGF1α (6,15-dk-dh-, 6-keto-, d17 6-keto-) Rv (D1, E1) tetranor (12-HETE, PGDM) TX (B1, B2, B3, 12-dehydro-TXB2, 2,3-dinor-TXB2)	human plasma	SPE	HSS T3 2.1 × 100 mm, 1.8 μm	A: 0.1% FAc in H_2_O B: 0.1% FAc in ACN	LC–MS/MS	QQQ	0.24–156.25 pg	[84]

ACN: Acetonitrile; CSF: Cerebrospinal fluid; EA: Ethyl acetate; FAc: Formic acid; HAc: Acetic acid; LOD: Limit of detection; LOQ: Limit of quantification; LLOQ: Lower limit of quantification; MeOH: Methanol; Q: Quadrupole; Q-IM-TOF: Quadrupole-ion mobility-time of flight; QQQ: Triple quadrupole; QQQ-TOF: Triple quadrupole-time of flight.
